# Genome-wide Association Studies of REST Gene Associated Neurological Diseases/traits with Related Single Nucleotide Polymorphisms

**DOI:** 10.2174/1567202620666230727153306

**Published:** 2023

**Authors:** Jingjing Wang, Sagor Kumar Roy, Seidu A. Richard, Yuming Xu

**Affiliations:** 1 Department of Neurology, The First Affiliated Hospital of Zhengzhou University, Zhengzhou University, Henan, Zhengzhou, 450000, P.R. China;; 2 Institute of Neuroscience, Third Affiliated Hospital of Zhengzhou University, Zhengzhou University, Zhengzhou, 450052, China;; 3 People’s Hospital of Luanchuan, Henan, 471599, Luoyang, P.R. China;; 4 The Academy of Medical Sciences of Zhengzhou University, Henan, 450000, Zhengzhou, P.R. China

**Keywords:** GWAS, REST, NRSF, ADHD, cognitive dysfunction, migraine disorder

## Abstract

**Background:**

Genome-wide association studies (GWAS) have been used to explore the connections between genotypes and phenotypes by comparing the genotype frequencies of genetic changes in individuals with similar origins but distinct traits.

**Objectives:**

The aim is to employ the GWAS catalog to identify and investigate the various correlations between genotypes and phenotypes of the REST gene.

**Methods:**

In this study, we utilized a large dataset of GWAS comprising 62,218,976 individuals in 112 studies and 122 associations with 122 traits (www.ebi.ac.uk/gwas/genes/REST) from European, Asian, Hispanic, African ancestry up to 28 February 2023. Protein-association network evaluation and gene ontology enrichment study was utilized to evaluate the biological function of the discovered gene modules.

**Results:**

We identified several associations for both neurodevelopmental and neurodegenerative disorders linked to REST, as well as its mapped gene modules and their functional relationship networks.

**Conclusion:**

This work offers fresh insights into identifying risk loci of neurological disorders caused by REST.

## 1 INTRODUCTION

Genome-wide association studies (GWAS) explore the connections between genotypes and phenotypes by comparing the genotype frequencies of genetic changes in individuals with similar origins but distinct traits. While single-nucleotide polymorphisms (SNPs) are the most commonly investigated genetic variants in GWAS, copy-number variants and sequence changes in the genetic code may also be considered. Numerous repeated genetic risk sites have been linked to diseases and phenotypes following 15 years of GWAS [1]. In epidemiological studies, trait-associated genetic variants, for example, may be used as control variables to account for influencing genetic group differences [2].

The Repressor Element 1 Silencing Transcription factor (REST) is a transcription controller with an alternative name such as neuron restrictive silencer factor (NRSF). It binds to a specific 21-bp motif known as Repressor Element 1-RE1 in the promoter region of genes encoding [3, 4]. REST functions as a negative regulator of neural gene expression throughout both embryogenesis and adult neurogenesis [5, 6]. The ease of access to certain DNA binding elements, the binding modes, and the collaboration and rivalry with other transcription elements are some of the variables that determines how REST affects its possible target genes [7-9]. When REST binds to DNA, it serves as a framework building and placing its functional complexes, which comprise of crucial enzymes like the demethylase LSD1 and histone deacetylases, among others. These complexes alter crucial histone and DNA locations to suppress the transcription of a significant number of genes [10-12].

In this research, we conducted an integrated network-based evaluation of GWAS and REST gene expression patterns of related neurological disorders. We speculated that conducting a meta-analysis on GWAS data and REST could reveal gene expression patterns of various neurological disorders. We first carried out a detailed search of REST linked neurological disease modules. After that, protein-association network evaluation and gene ontology (GO) enrichment study were utilized to evaluate the biological function of the discovered gene modules. Additionally, we demonstrated how well multi-omics data were integrated and mined for research on neurological illnesses related to REST.

## 2 MATERIALS AND METHODS

### 2.1 Genome-wide Association and Single-nucleotide Polymorphisms Analysis

**Fig. (1):**
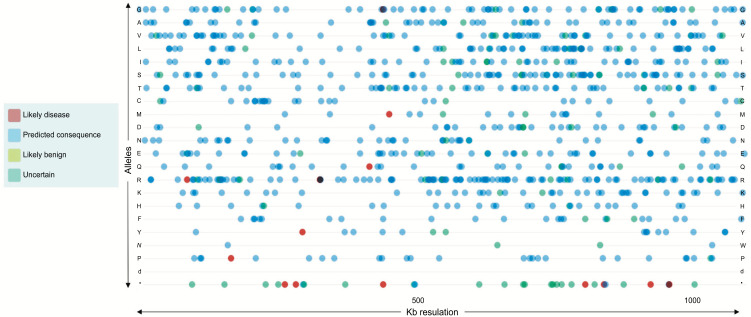
Variant SNPs with Neurological disease/traits showing with genomic position from 0 to 1097 kb resolution with their -log10 p-values.

We utilized a large dataset of GWAS catalog comprising of 62,218,976 individuals including cases and controls in 112 studies and 122 associations with 122 traits (www.ebi.a c.uk/gwas/genes/REST) from European, Asian, Hispanic, African ancestry up to 28 February, 2023. All these data enriched various variants of SNP and risk allele for their disease or trait Genome-wide association study (GWAS) and SNPs analysis from https://platform.opentargets.org/ (Fig. **1**). Our large sample were analyzed using risk allele frequencies (RAF), odd ratios (OR), 95% clearance values and also their p-values. Using pairwise identity testing by condition, a genetic relatedness check was successfully conducted for all study samples [13].

Genetically unrelated subjects as well as SNPs with call rates 95.00% (99% for WTCCC GWAS data), minor risk allele frequencies 0.01 or Hardy-Weinberg Equilibrium testing with p-values of 10^-6^ (10^-5^ for WTCCC GWAS data) were excluded. The meta-analysis was conducted by Microsoft excel 16.54 version (21101001). The final results with genetic control correction were tabulated. The original studies contain thorough details of the study subjects, innovative methods, and analytical strategies. The overall outcome of results of *p*-value were sorted out by Fisher's exact method and < 0.05 was considered as significant.

### 2.2 REST and Neurological Disease Analysis

We selected only probable neuro-psychiatric diseases or traits totaling 464,915 individuals with European ancestry in 3 cohort studies with more than thousand imputed genotyped SNPs /variants and risk allele for meta-analysis. All these data were collected from “EMBL's European Bioinformatics Institute (EMBL-EBI)” -the potential of big data of biology, UK (www.ebi.ac.uk/gwas/genes/REST). The association analysis was performed using linear regression for each GWAS according to diseases or traits which included their SNPs allele, chromosome position, and mapped genes. Furthermore, OR and 95% clearance values of all SNPs including their diseases or traits are gathered and input on Microsoft excel 16.54 version. Multiple tables and forest plots were conducted according to the data collected from different neurological diseases or traits.

## 3 RESULTS

### 3.1 Genome-wide Association and Single-nucleotide Polymorphisms Analysis of REST and Neurological Diseases/traits

**Fig. (2):**
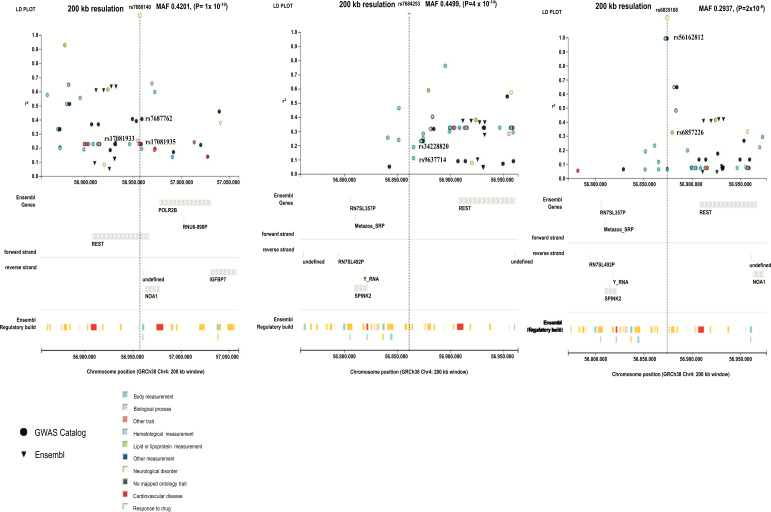
Regional plots or Linkage disequilibrium (LD) plot of association results and recombination rates for newly identified risk loci rs7680140, rs7684253 and rs6835108 with their minor allele frequency (MAF) values and nearest SNP variants. Loci are shown at both 200 kb resolutions. ChIP transcription factor binding sites shown as grey bars. GWAS pane shows plots show association -log10 *P*-values (left y-axis) of SNPs shown according to their chromosomal positions (x-axis).

In a discovery sample comprising of 112 studies, we found different neurological disease/trait with their SNPs from individuals of European descent. SNP rs7680140-A represents attention deficit hyperactivity disorder (ADHD), with antisocial behavior measurement and substance abuse with odd ratio (OR), 95% clearance value (95% CI) = 0.007 (0.005-0.009), (*P* = 1 x 10^-10^) following alleles A/G (forward strand), with minor allele G and its frequency 0.4201. Other nearest SNPs are rs17081935, rs17081933 and rs7687762 showed body measurement, coronary artery disease and myocardial infarction with r2 value 0.233727. SNP rs7684253-T is related to migraine disorder with OR, 95% CL=1.03999, (1.03-1.05), (*P* = 4 x 10^-14^) presenting alleles C/T (forward strand, with minor allele T and its frequency 0.4499. The linkage disequilibrium (LD) plot showed closest SNPs are rs34228820 and rs9637714 with r^2^ values are 0.195226 and 0.118457. SNP rs6835108-A is linked to unipolar depression with cognitive dysfunction (*P* = 2x10^-8^), minor allele A/G (forward strand), minor allele G and its frequency 0.2937. Nearest SNPs rs56162812 are related to mean platelet count with r^2^ value 0.069072 and rs6857226 is related to formation of cortical morphology (*P* =4.00 × 10^-8^), allele G/A (forward strand), minor allele A, and its frequency 0.2937, r^2^ value 1.00. The overall *p*- value is sorted out by Fisher's exact method and *p*-value is 4x10^-14^. rs7680140-A is solely mapped with REST gene and other SNPs are mapped together with SPINK2 and REST genes and all SNPs have intergenic variant feature (Table **1** and Fig. **2**).

### 3.2 Analysis of SNPs of REST Related Neurological Diseases/traits

**Fig. (3):**
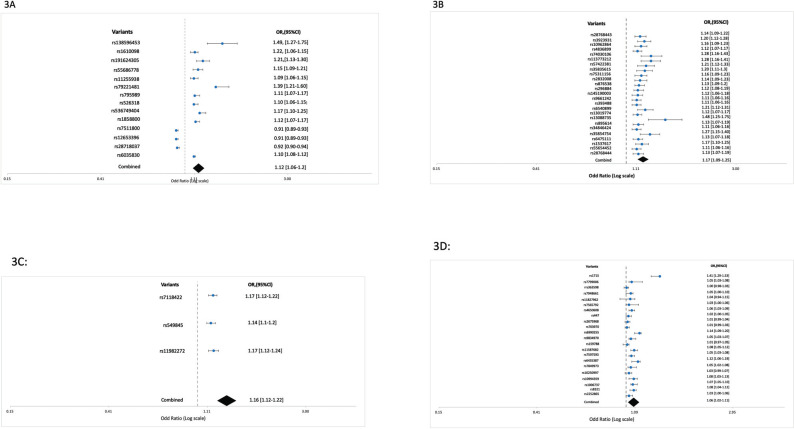
REST gene associates diseases: (**A**) REST gene associates with Childhood Attention deficit hyperactivity disorder (ADHD) with their combining values of odd ratio and 95% clearance confidence level. (**B**) REST gene associates with Persistent Attention deficit hyperactivity disorder (ADHD) with their combining values of odd ratio and 95% clearance confidence level. (**C**) REST gene associates with Disruptive behavior disorder with attention deficit hyperactivity disorder (ADHD) with their combining values of odd ratio and 95% clearance confidence level. (**D**) REST gene associates with autism spectrum disorder with attention deficit hyperactivity disorder (ADHD) with their combining values of odd ratio and 95% clearance confidence level.

We specifically divided ADHD into several traits associated with childhood and persistent ADHD, ADHD with disruptive behavior disorder, autism spectrum disease. A total of 14 variants/SNPs are observed with childhood ADHD with forest plot showed combined OR and 95% clearance value is 1.12 [1.06-1.^20^] (Fig. **3A**) and overall p-value was 1.81 × 10^-6^. The corresponding mapped genes with chromosomal positions and risk adjustment factor (RAF) are shown in Table **2A**. We found 25 variants with their mapped genes along with chromosomal position and RAF for Persistent ADHD (Table **2B**). The forest plot calculation revealed combined OR and 95% clearance value is 1.17 [1.09-1.^25^] and overall p-value is 1.76 × 10^-5^ (Fig. **3B**).

Only 3 variants are found for disruptive behavior with ADHD and combined OR and 95% clearance value showed 1.16 [1.12-1.^22^] and overall p-value of 3 × 10^-10^ (Fig. **3C**). The mapped gene and RAF were shown in Table **2C**. We also sorted out 22 variants of autism spectrum disorder (ASD) with ADHD (Modelling analysis) including their mapped genes and RAF (Table **2D**). The forest plot reveals combined OR and 95% clearance value 1.06 [1.02-1.^11^] and overall *p*-value 8.4022 × 10^-8^ (Fig. **3D**). ADHD with Bipolar disorder and schizophrenia related mapped gene with their p-values are shown in Tables **2E** and **F**.

We have found 7 variants of cognitive impairment itself and their mapped genes, chromosome positions and RAF are shown in Table **3A**.

**Fig. (4):**
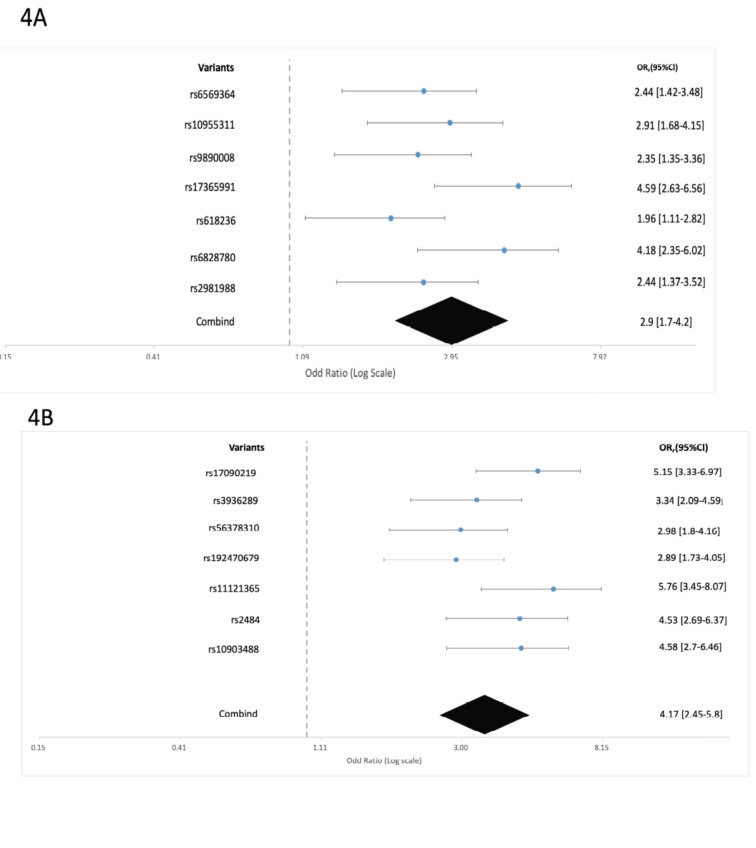
Variants associates with odd ratio and 95% clearance confidence level. (**A**). Variants associates’ Cognitive impairment with their combining values of odd ratio and 95% clearance confidence level. (**B**). Variants associates’ Cognitive impairment as Alzheimer’s Disease (AD) with their combining values of odd ratio and 95% clearance confidence level.

The forest plot showed combined OR and 95% clearance value of cognitive impairment itself is 2.9 [1.7-4.^2^] and overall *p*-value of 1.9 × 10^-5^ (Fig. **4A**). Moreover, 7 variants were identified for cognitive impairment towards AD with mapped genes with chromosome position with RAF are shown in Table **3B**.

The combined odd ratio and the 95% confidence revealed 4.17 [2.45-5.^8^] and ultimate p-value was sorted out as 4.59 × 10^-6^ (Fig. **4B**).

Besides, four varieties of SNP mutations in migraine patients with subjective cognitive impairment were evaluated. A significant association among SNPs and clinical indicators in migraineurs with cognitive impairment with mapped genes and RAF was shown in Table **4**.

**Fig. (5):**
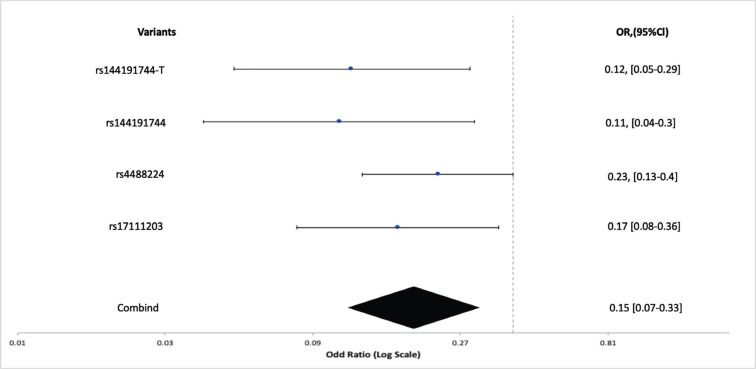
Variants associates’ Migraine with cognitive decline with their combining values of odd ratio and 95% clearance confidence level.

The combined result of the OR and the 95% confidence intervals showing 0.15 [0.07-0.^33^] in forest plot of migraineurs and overall *p*-value was 3.04 × 10^-8^ (Fig. **5**).

Using case-only analysis, we found no connection between sex or proper age at ADHD, cognitive dysfunction and migraine diagnosis with the novel risk SNPs. Using GWAS catalog data, we discovered no statistically significant association between SNP genotype and patient outcome. Yet, an inability to establish further correlations may be a result of insufficient statistical power.

## 4 DISCUSSION

As shown by GWAS, genomic risk sites are correlated SNPs that are statistically strongly connected with the variable of interest. Numerous applications can be considered utilizing GWAS findings. This research combines bioinformatic and biochemical methods to identify RE1 locations and REST target genes across the whole genome. We carried out a network-based integrative analysis of GWAS in this study and found 122 associates with 122 traits for sites of REST. It is significant to highlight that we have discovered a number of neurological diseases connected with REST as well as the connected genes, gene clusters, and network connections.

The significance of processing and presentation of antigens in the etiology of these disorders is underlined by the study of functional enrichment of defined gene modules. To reduce the number of false positives in this study, it was thought crucial to use a conservative consensus sequence. Additionally, because certain bases that were recognized as essential in the framework of one consensual transcriptional regulator have been shown to be redundant in the framework of other regulatory components, adoption of a too strict consensus could result in the deletion of legitimate targets [14].

In addition, we propose functional association networks of REST-associated disorders and their gene modules, which may assist in elucidating the complicated aetiology of these diseases. Further research is required to corroborate our results. The REST gene is related with not only neurodevelopmental such as ADHD but also it is associated with ASD, psychiatric disorder such as bipolar disorder and schizophrenia [15]. It also is related to neurodegenerative such as cognitive impairment and as AD and subjective cognitive impairment with migraine headache [16]. Meanwhile, other mapped genes were exposed to these diseases which could be directly or indirectly related to REST.

Earlier, it was hypothesized that the promoter and other regulatory elements of the REST target genes comprised between one and five RE-1s (also termed as neuron-restrictive silencer elements), which are the REST-binding DNA sequences. There were believed to be approximately a thousand RE-1-positive genes, the majority of which coded for nerve cell proteins [17-19]. REST/NRSF contains two repressor domains following amino and carboxyl termini, respectively and a core DNA-binding region with eight parallel C2H2 Zinc Fingers (ZFs) [3, 20, 21]. Numerous NRSEs that belong into the categories of canonical, noncanonical, and half-site only motifs have been demonstrated to bind to REST/NRSF [22]. A notable difference between canonical and noncanonical NRSEs is the extent of the distance separating the left- and right-halves. There are more than 800 genes in the human genome that code for ZF transcription factors, which are tiny DNA-recognition units that are typically grouped in tandem [23-25].

The bulk of REST research to far have focused on neural stem cells, embryonic stem cells, and brain cells in the process of differentiation [26, 27]. High levels of REST have been demonstrated to suppress a significant number of genes in embryonic stem cells when combined with other aspects, including the conventional pluripotency proteins Sox2, Oct4, and Nanog [8]. REST has already been demonstrated to enhance gene expression in addition to repression, which has been observed at all phases of cell differentiation [12, 28]. Because of its capacity for interaction with other variables, such as those controlled through the Polycomb complexes, REST can play a dual role in transcriptional regulatory networks [12, 28]. Although chromatin alterations are essential for transcriptional remodeling during the process of cellular differentiation, it’s indeed not yet known how they are directed to certain positions.

REST quickly falls to extremely low levels when neuronal development rapidly advances to a mature progenitor phase. This is partly a result of two crucial regulators, HIPPI (HIP1 protein interactor) and beta-catenin, exerting less control [29, 30]. REST’s downregulation state is responsible for the development and maintenance of neuronal specializations. During advanced differentiation, the activation of REST-dependent genes drives a number of crucial processes, such as axonal development, the creation of synaptic connections, and membrane excitability [31, 32]. Although adult neurons have a low level of REST, the average content in brain tissue is significant. In actuality, the majority of nonneuronal glial cells have high REST levels [33], in neural stem cells clustered specifically in some regions such as subventricular zones, dentate gyrus of the hippocampus, and several others where neurogenesis occurs, as well as in endothelium and other vessel cells [6].

Several of the following research confirmed and enhanced the concept proposed by Chong *et al.* (1995) through their finding article that modest amounts of REST are required to permit the transcription of neural genes in neurons [3]. REST participates in chromatin plasticity *via* regulating gene expression [5, 7]. REST controls or has connections to several genes which are expressed in neuronal maturation. These include transcription factor genes like, Grin1, Sp1, Ascl1, Isl1, and few others that depend on REST for their repression in addition to genes that are not suppressed. REST affects the functioning of several genes, which include E47, Poh3f2, Creb, Gata2, Myod, and many more [27, 34-36].

Transmitter release is among the neuron-specific activities regulated through REST. In this instance, REST inhibits the genes responsible for vesicle exocytosis and those that code for synaptic vesicle proteins. These proteins consist of specific SNAREs, neurotransmitter transporters, as well as other membrane proteins, in addition to proteins aggregated in the lumen or exposed on the cytosolic surface of vesicles, including the most abundant protein, synapsin 1, which is essential for vesicle traffic and recycling [20, 37, 38]. For REST physiology to function, other processes are required, including as the factor's trafficking to the nucleus. The concentration of the repressor in the nucleus, where its effect is contained, is the procedure that has been studied in detail [39]. However, different disorders, including Huntington’s disease and Alzheimer's disease (AD), impair REST transport in different ways such as stimulation [40]; and depression [41]. REST levels play a crucial role in neuronal specificity by controlling elements involved in alternative mRNA splicing. For example, REST regulates nSR100's expression, which affects a wide range of mRNAs, which are alternatively neurons and non-neural cells are spliced in [42]. One of these is the mRNA for REST, commonly manifested as the inactive, shortened version REST4, which interacts with a full-length form of REST for target gene binding in neurons. Existence of both the full-length and shortened forms is noteworthy because it reduces the suppression of REST regulated genes and preserves neuronal cells [42].

Through the reciprocal complementing and validation of diverse GWAS data, integrative studies of SNPs association have the ability to decrease the impact of data inaccuracy and statistical prejudice on study outcomes. Our data reveals three connections between the REST gene and the risk of acquiring neurological diseases. In addition to offering additional evidence for genetic vulnerability to ADHD, cognitive dysfunction, and migraine disorder, these novel risk loci also shed light on the molecular basis. It provides substantial benefits for demonstrating the actual concepts and patterns of worldwide pathogen activity of complicated disorders when combined with this research. However, GWAS study methodologies and tools are not widely available, thus they need more research.

## CONCLUSION

Finally, network-based integrative analysis of GWAS of RE1s reveals possible neurological disorders and probable REST-targeting genes. We identified various target gene groups according to their occupancy and REST regulation through integrating this data into a freely accessible online database. According to type of cells and/or stage of development, we predict that membership in these groupings will change. We discovered a number of associations for both neurodevelopmental and neurodegenerative disorders linked to REST, as well as its mapped gene modules and their functional relationship networks. This work offers fresh insights into the identification of associations of neurological disorders caused by REST.

## Figures and Tables

**Table 1:** REST gene associates variants and risk allele Neurological diseases attention deficit hyperactivity disorder (ADHD), Migraine disorder, unipolar depression with cognitive dysfunction with their odd ratio (OR) and 95% clearance value with *P*-values.

**Variant SNP and Risk Allele**	**Chromosome: Position**	**RAF**	**Diseases/traits**	**(OR (95% CI)**	***P*-value**	**Mapped Gene**	**Feature**
rs7680140-A	4:56957291	0.531	ADHD, substance abuse, antisocial behaviors	0.007, (0.005-0.009)	1 x 10^-10^	REST	Intron variant
rs7684253-T	4:56861145	0.55	Migraine Disorder	1.03999, (1.03-1.05)	4 x 10^-14^	SPINK2- REST	Intergenic variant
rs6835108-A	4:56874168	0.77	Unipolar depressive disorder with cognitive dysfunction	-	2 x 10^-8^	SPINK2- REST	Intergenic variant

**Table 2A:** Variant and risk allele, risk adjustment factor (RAF), map genes, location and *p*-value with childhood attention deficit hyperactivity disorder (ADHD).

**Variant and Risk Allele**	**RAF**	**Mapped Gene**	**Location**	***P*-Value**
rs138596453-<b>?</b>	0.98	GAB4	22:17006586	6 x 10-7
rs1610098-<b>?</b>	0.37	SEMA6D	15:47513815	8 x 10-7
rs191624305-<b>T</b>	0.9	TNR	1:175353170	2 x 10-7
rs55686778-<b>T</b>	0.19	TMEM114	16:8458589	2 x 10-7
rs11255938-<b>?</b>	0.54	LINC02676	10:8808772	2 x 10-6
rs79221481-<b>T</b>	0.03	EPCAM-DT	2:47277861	3 x 10-6
rs795989-<b>A</b>	0.68	MAML3	4:139808192	2 x 10-6
rs526318-<b>T</b>	0.54	RNA5SP214	6:117070716	3 x 10-6
rs536749404-<b>T</b>	0.14	-	8:13806960	3 x 10-6
rs1858800-<b>T</b>	0.35	ZFHX3	16:72990377	1 x 10-6
rs7511800-<b>T</b>	0.31	ST3GAL3	1:43748598	7x 10-11
rs12653396-<b>T</b>	0.42	LINC00461	5:88551455	2x 10-11
rs28718037-<b>A</b>	0.33	DCC	18:53046327	9 x 10-9
rs6035830-<b>C</b>	0.28	XRN2, ZNF877P	20:21285090	2 x 10-9

**Table 2B:** Variant and risk allele, risk adjustment factor (RAF), map genes, location and *P* value with persistent attention deficit hyperactivity disorder (ADHD).

**Variant and Risk Allele**	**RAF**	**Mapped Gene**	**Location**	***P*-Value**
rs28768443-<b>?</b>	0.81	-	14:29285390	6 x 10^-7^
rs3923931-<b>A</b>	0.11	CNBD1	8:87401743	2 x 10^-7^
rs10962864-<b>T</b>	0.16	RPS29P33, RN7SL720P	9:17091466	5 x 10^-7^
rs4836899-<b>T</b>	0.52	ASTN2	9:116894150	3 x 10^-7^
rs74030106-<b>?</b>	0.05	TMC3-AS1	15:81525290	2 x 10^-6^
rs113773212-<b>?</b>	0.05	LINC00917	16:86361158	2 x 10^-6^
rs57422381-<b>?</b>	0.07	LINC00456, MBNL2	13:97205239	3 x 10^-6^
rs35835615-<b>?</b>	0.09	SPCS2P2, ERLIN1	10:100145181	1 x 10^-6^
rs75311156-<b>?</b>	0.82	OR5H3P, OR5H5P	3:98198801	4 x 10^-6^
rs2832008-<b>?</b>	0.14	U6	21:28737676	4 x 10^-6^
rs876538-<b>?</b>	0.79	CRPP1, CRP	1:159705927	2 x 10^-6^
rs296884-<b>?</b>	0.74	HNRNPK, C9orf64	9:83968008	1 x 10^-6^
rs145190003-<b>?</b>	0.26	CNTLN	9:17263750	3 x 10^-6^
rs9661242-<b>?</b>	0.48	-	1:29615908	2 x 10^-6^
rs393488-<b>?</b>	0.53	BNC2, RN7SL720P	9:17044973	3 x 10^-6^
rs6540899-<b>A</b>	0.12	KCTD3	1:215479116	2 x 10^-6^
rs13019774-<b>T</b>	0.39	GALNT13, RNA5SP107	2:154505440	1 x 10^-6^
rs13088735-<b>T</b>	0.02	LMCD1-AS1	3:8123815	3 x 10^-6^
rs895614-<b>A</b>	0.29	RNU6-276P	4:55792612	4 x 10^-6^
rs34846424-<b>T</b>	0.36	LINC02374	4:187244864	4 x 10^-6^
rs35854754-<b>CT</b>	0.94	-	6:91356930	3 x 10^-6^
rs6475111-<b>T</b>	0.26	CNTLN	9:17162415	5 x 10^-6^
rs1537617-<b>A</b>	0.84	-	10:2740611	2 x 10^-6^
rs55654452-<b>G</b>	0.56	ZNF521	18:25064312	2 x 10^-6^
rs35782676-<b>T</b>	0.77	ZNF584	19:58414978	4 x 10^-6^

**Table 2C:** Variant and risk allele, risk adjustment factor (RAF), map genes, location and *P* value with Disruptive behavior disorder & attention deficit hyperactivity disorder (ADHD).

**Variant and Risk Allele**	**RAF**	**Mapped Gene**	**Location**	***P*-Value**
rs7118422-<b>T</b>	0.51	STIM1	11:3912065	3x 10^-10^
rs549845-<b>G</b>	0.30	PTPRF	1:43610798	2 x 10^-7^
rs11982272-<b>T</b>	0.78	MAD1L1	7:1931130	3 x 10^-7^

**Table 2D:** Variant and risk allele, risk adjustment factor (RAF), map genes, location and *P* value with autism spectrum disorder (ASD) & attention deficit hyperactivity disorder (ADHD) (Modelling analysis).

**Variant and Risk Allele**	**RAF**	**Mapped Gene**	**Location**	***P*-Value**
rs2021722-<b>?</b>	0.78	TRIM26	6:30206354	2x 10^-12^
rs10503253-<b>?</b>	0.19	CSMD1	8:4323322	4 x 10^-8^
rs17662626-<b>?</b>	0.91	-	2:193119895	5 x 10^-8^
rs548181-<b>?</b>	0.88	STT3A, FEZ1	11:125591814	9 x 10^-7^
rs2799573-<b>?</b>	0.71	CACNB2	10:18312999	4 x 10^-8^
rs1625579-<b>?</b>	0.80	MIR137HG	1:98037378	2x 10^-11^
rs1480380-<b>C</b>	0.90	HLA-DMA, HLA-DMB	6:32945469	6 x 10^-7^
rs12966547-<b>?</b>	0.58	LINC01929	18:55084786	3x 10^-10^
rs7914558-<b>?</b>	0.58	CNNM2	10:103016151	2 x 10^-9^
rs11191580-<b>?</b>	0.91	NT5C2	10:103146454	1 x 10^-8^
rs7004633-<b>?</b>	0.18	-	8:88748082	3 x 10_-8_
rs9371601-<b>?</b>	0.34	SYNE1	6:152469438	4 x 10^-9^

**Table 2E:** Variants associates mapped gene variant and risk allele, location and *p*-value with attention deficit hyperactivity disorder (ADHD) & bipolar disorder.

**Variant and Risk Allele**	**Mapped Gene**	**Position**	***P*-Value**
rs58502974-<b>A</b>	ADCY2	5:7755787	2 x 10^-8^
rs1334489-<b>?</b>	SLC35F1	6:118318224	7 x 10^-6^
rs2134095-<b>?</b>	RXRG	1:165408315	7 x 10^-6^
rs16998572-<b>?</b>	SUMO1P1, BCAS1	20:53935345	8 x 10^-6^
rs11162556-<b>?</b>	ADGRL4	1:78795698	2 x 10^-6^
rs12761122-<b>?</b>	LINC02626, TRUB1	10:114993789	4 x 10^-6^
rs7089973-<b>C</b>	TAF9BP2	10:114809806	2 x 10^-8^
rs11756438-<b>A</b>	CEP85L	6:118672469	4 x 10^-8^
rs34096808-<b>?</b>	NLGN1	3:173822316	1 x 10^-7^
rs58502974-<b>?</b>	ADCY2	5:7755787	1 x 10^-7^
rs10489744-<b>?</b>	RXRG	1:165411386	2 x 10^-7^
rs9608816-<b>?</b>	ASCC2	22:29820911	6 x 10^-7^
rs16998572-<b>?</b>	SUMO1P1, BCAS1	20:53935345	8 x 10^-7^
rs36030485-<b>?</b>	VLDLR-AS1	9:2411646	9 x 10^-7^
rs11162556-<b>?</b>	ADGRL4	1:78795698	4 x 10^-7^
rs56037433-<b>?</b>	SLC4A10	2:161719475	9 x 10^-7^
rs71420669-<b>?</b>	'-	2:194986941	9 x 10^-7^
rs13061878-<b>?</b>	PTPRG	3:61634560	8 x 10^-7^
rs1334489-<b>?</b>	SLC35F1	6:118318224	6 x 10^-8^
rs12661338-<b>?</b>	CEP85L	6:118473527	5 x 10^-7^
rs968847-<b>?</b>	LINC02626, ATRNL1	10:114998320	3 x 10^-7^

**Table 2F:** Variants associate mapped gene Variant and risk allele, location and *P* value with attention deficit hyperactivity disorder (ADHD) & Schizophrenia.

**Variant and Risk Allele**	**Mapped Gene**	**Position**	***P*-Value**
rs12025137-<b>?</b>	LINC01787, UBE2WP1	1:96417671	8 x 10^-10^
rs9659624-<b>?</b>	MIR137HG	1:98021777	8 x 10^-14^
rs79366331-<b>?</b>	MINDY1	1:150998719	3 x 10^-8^
rs10802742-<b>?</b>	-	1:239038351	3 x 10^-8^
rs12058508-<b>?</b>	SDCCAG8	1:243324559	3 x 10^-8^
rs57159364-<b>?</b>	ZNF804A	2:184643769	2 x 10^-9^
rs1451488-<b>?</b>	RNU7-147P	2:199125384	2 x 10^-11^
rs796364-<b>?</b>	FTCDNL1, RN7SL717P	2:199851396	5 x 10^-10^
rs6704768-<b>?</b>	GIGYF2	2:232727791	3 x 10^-17^
rs9882879-<b>?</b>	LINC02033, HSPD1P6	3:36802580	1 x 10^-9^
rs1080500-<b>?</b>	RFT1, PRKCD	3:53141001	2 x 10^-8^
rs9845457-<b>?</b>	MSL2	3:136195634	2 x 10^-8^
rs13135092-<b>?</b>	SLC39A8	4:102276925	4 x 10^-8^
rs223346-<b>?</b>	UBE2D3	4:102859339	8 x 10^-10^
rs62367473-<b>?</b>	HCN1	5:45216147	2 x 10^-8^
rs34635-<b>?</b>	LINC02057, SMIM15-AS1	5:61217674	2 x 10^-8^
rs13361438-<b>?</b>	LINC01470	5:152627515	1 x 10^-8^
rs12668848-<b>?</b>	MAD1L1	7:1981360	8 x 10^-11^
rs42694-<b>?</b>	CREB5	7:28389624	8 x 10^-9^
rs56150095-<b>?</b>	CALN1	7:72294084	5 x 10^-12^
rs12704290-<b>?</b>	GRM3, GRM3-AS1	7:86798310	2 x 10^-10^
rs12539177-<b>?</b>	IMMP2L	7:111337645	3 x 10^-10^
rs10954580-<b>?</b>	DGKI	7:137391966	1 x 10^-8^
rs1473594-<b>?</b>	-	8:59783967	1 x 10_-8_
rs4129585-<b>?</b>	TSNARE1	8:142231572	4 x 10^-12^
rs295268-<b>?</b>	GKAP1	9:83814390	6 x 10^-10^
rs713240-<b>?</b>	ARID5B	10:62050693	5 x 10^-9^
rs12244388-<b>?</b>	BORCS7-ASMT, AS3MT	10:102880295	4 x 10^-8^
rs4298967-<b>?</b>	CACNA1C	12:2299028	1 x 10^-11^
rs324017-<b>?</b>	NAB2	12:57094031	3 x 10^-9^
rs704061-<b>?</b>	POC1B, DUSP6	12:89378126	2 x 10^-8^
rs3764002-<b>?</b>	WSCD2	12:108224853	3 x 10^-10^
rs2102949-<b>?</b>	MPHOSPH9	12:123192216	2 x 10^-13^
rs11844846-<b>?</b>	LINC02326	14:29018462	4 x 10^-8^
rs8009147-<b>?</b>	PPP1R13B	14:103798325	5 x 10^-9^
rs12592235-<b>?</b>	SEMA6D	15:47495773	1 x 10^-8^
rs12898315-<b>?</b>	-	15:61561804	1 x 10^-10^
rs4925114-<b>?</b>	RAI1	17:17807956	3 x 10^-9^
rs17594665-<b>?</b>	TCF4	18:55396488	6 x 10^-9^
rs2053079-<b>?</b>	ZNF536	19:30496516	3 x 10^-8^
rs603985-<b>?</b>	FUT2	19:48704000	3 x 10^-8^
rs2263655-<b>?</b>	RNU6-917P, SIRPB2	20:1527453	8 x 10^-10^
rs6071524-<b>?</b>	PPP1R16B, ACTR5	20:38797937	3 x 10^-11^

**Table 3A:** Variant and risk allele associates with mapped gene with Cognitive impairment. Variant and risk allele, risk adjustment factor (RAF), map genes, location and *P* value with cognitive impairment.

**Variant SNP**	**RAF**	**Mapped Gene**	**Region**	**Location**	***P*-Value**
rs6569364	0.22	NKAIN2	6q22.31	123837107	4.00E-06
rs10955311	0.12	BAALC-AS1, BAALC	8q22.3	103166349	5.00E-06
rs9890008	0.25	-	17p12	14568376	5.00E-06
rs17365991	-	TEF	22q13.2	41394175	0.000005
rs618236	-	RIT2	18q12.3	42754135	0.000009
rs6828780	-	SHROOM3-AS1- SOWAHB	4q21.1	76878696	0.000009
rs2981988	-	TTLL2, TCP10L3	6q27	167357248	0.00001

**Table 3B:** Variants associates mapped genes with Cognitive impairment as AD. Variant and risk allele, risk adjustment factor (RAF), map genes, location, and *P* value with cognitive impairment as Alzheimer’s Disease (AD).

**Variant SNP**	**Mapped Gene**	**Region**	**Location**	***P*-Value**
rs17090219	NA - TXNL1	18q21.31	56523802	0.00000009
rs3936289	ST6GAL1	3q27.3	103166349	0.0000005
rs56378310	RAB20	13q34	110537326	0.000002
rs192470679	PDS5B	13q13.1	32716722	0.000002
rs11121365	SPSB1	1p36.22	9297665	0.000002
rs2484	BDH1	3q29	197541698	0.000003
rs10903488	ADARB2	10p15.3	1563803	0.00001

**Table 4:** Variants associate mapped gene migraine disorder with cognitive decline. SNP-RISK ALLELE with mapped gene with *P* values of migraine disorder related to cognitive decline.

**Strongest SNP-Risk Allele**	**Position**	**Mapped Gene**	***P*-Value**
rs144191744-T	1p22.1	TGFBR3	3.00E-08
rs144191744-?	1p22.1	TGFBR3	4.00E-10
rs4488224-?	11q14.2	PSMA2P1 - RNU6-1135P	1.00E-07
rs17111203-?	1p22.1	ARHGAP29	6.00E-07

## Data Availability

The data used in this paper is publicly available and can be searched in the website available in the method section of the paper.

## LIST OF ABBREVIATIONS

GO: Gene Ontology
GWAS: Genome-wide Association Studies
NRSF: Neuron Restrictive Silencer Factor
SNPs: Single-nucleotide Polymorphisms
